# Violence Against Administrators: The Roles of Student, School, and Community Strengths and Cultural Pluralism

**DOI:** 10.3390/bs15040556

**Published:** 2025-04-21

**Authors:** Susan D. McMahon, Andrew H. Perry, Taylor Swenski, Kailyn Bare, Jared Hunt, Andrew Martinez, Linda A. Reddy, Eric M. Anderman, Ron Avi Astor, Dorothy L. Espelage, Frank C. Worrell, Christopher M. Dudek

**Affiliations:** 1 Department of Psychology, DePaul University, Chicago, IL 60614, USA; tswenski@depaul.edu (T.S.); kbare1@depaul.edu (K.B.); jhunt30@depaul.edu (J.H.); 2 Department of Educational Studies, The Ohio State University, Columbus, OH 43210, USA; perry.2221@buckeyemail.osu.edu (A.H.P.); anderman.1@osu.edu (E.M.A.); 3 New York Center for Justice Innovation, New York, NY 10018, USA; anmartinez@nycourts.gov; 4 Graduate School of Applied and Professional Psychology, Rutgers University, New Brunswick, NJ 08901, USA; lreddy@rutgers.edu (L.A.R.); cdudek@gsapp.rutgers.edu (C.M.D.); 5 Social Welfare and Education, University of California-Los Angeles, Los Angeles, CA 90095, USA; astor@luskin.ucla.edu; 6 School of Education, University of North Carolina at Chapel Hill, Chapel Hill, NC 27599, USA; espelage@unc.edu; 7 Berkeley School of Education, University of California-Berkeley, Berkeley, CA 94720, USA; frankc@berkeley.edu

**Keywords:** school violence, administrator victimization, ecological model, student, parent, colleague violence, cultural pluralism

## Abstract

Scientific and public attention regarding educator-directed violence has increased over the past 15 years; however, research on violence against administrators is limited. Although school administrators are responsible for school performance and safety, they can be particularly vulnerable to violence from students, teachers, and parents. This study includes 497 pre-K–12th grade school administrators in the United States. A path analysis was conducted to examine the associations between administrator perceptions of student-, school-, and community-level strengths and administrator experiences of verbal/threatening and physical violence. Cultural pluralism, which incorporates student and staff support of cultural differences and honors different voices and cultures in curricula and discussion, was investigated as a moderator of these associations. Results indicate that (a) student strengths are associated with less student verbal/threatening violence against administrators; (b) school strengths are associated with less student and colleague verbal/threatening violence against administrators; and (c) community strengths are associated with less physical violence from students and less verbal/threatening violence from parents against administrators. Cultural pluralism practices significantly moderated the relationship between student strengths and physical violence from colleagues. Findings highlight school practices and policies across the school ecology that are associated with less administrator-directed violence.

## 1. Introduction

Violence directed against school personnel is a crisis of global scope (Botha & Zwane, 2021). Although most school violence research focuses on students and teachers (Longobardi et al., 2019), there is emerging evidence that administrators also experience violence in the workplace. A recent survey by the National Association of Secondary School Principals (NASSP, 2023) found that 70% of school leaders reported at least one verbal and threatening or physical violence incident during the 2021–2022 school year. About one-third of principals reported being threatened by parents and community members in response to their COVID-19 precautions (NASSP, 2021). McMahon et al. (2024) found that, in a national survey of the 2021–2022 school year, 30–77% of administrators reported experiencing verbal/threatening violence (e.g., threats, bullying, intimidation), while 2–43% reported physical violence (e.g., objects thrown, physical attacks), depending on the aggressor (student, parent, or colleague). Research in Ireland, New Zealand, and Australia also reveals high rates of violence against school administrators, ranging from 11 to 50% depending on the type of violence (Arnold et al., 2022; Dicke et al., 2024; Riley, 2015). In 2017, Australian prevalence rates of verbal and physical violence against administrators reached 38% and 44%, representing increases of 6% and 7%, respectively, from 2011 (Riley, 2018). As such, Australian school principals experience physical violence at rates 8.4 times higher than the general public in their country (Riley, 2018). Although rates are high, few empirical studies have examined administrator experiences across different types of violence (i.e., verbal, physical) and aggressors (i.e., students, parents, colleagues; Arnold et al., 2021). Further, there is a need to examine the ecological factors contributing to administrator-directed violence and how they may vary between types of violence and aggressor to inform practice and policy.

School administrators, including principals, vice principals, and superintendents, serve in high-pressure roles (Sogunro, 2012), with few mental health and stress reduction resources currently available to them (DeMatthews et al., 2021). Principals reported significant job-related stress, burnout, symptoms of depression, and inadequate coping abilities in the context of returning to school after COVID-19 (Steiner et al., 2022). Specifically, about half of principals reported stress due to staffing and supporting employee mental health and well-being, while about a third endorsed stress related to supporting student mental health and well-being, addressing COVID-19-related learning losses and COVID-19 mitigation strategies.

Administrators often report insufficient resources to effectively perform their jobs, including a lack of professional development and decision-making authority, insufficient salaries amidst demanding working conditions, teacher shortages, and high-stakes accountability measures which disincentivize retention in underperforming schools (Collie et al., 2020; Kaufman et al., 2022; NASSP, 2023). Nearly 40% of high school administrators report challenges with staff shortages (NASSP, 2023). Also, some administrators face backlash amidst increasing political polarization, which yielded bans and restrictions on critical race theory (CRT) and other identity-centered learning (Rogers et al., 2022).

Considering these workplace conditions, it is not surprising that there is a national shortage of principals and other administrators (Diliberti & Schwartz, 2023). The NASSP found that 38% of high school principals reported a desire to quit their jobs within the next three years (NASSP, 2023), suggesting further threats to the school leadership pipeline. Additionally, administrator concerns with school safety are associated with greater intentions to transfer schools (Perry et al., 2024). When administrators leave their positions, there are downstream consequences for students and staff, including lower student achievement, worse school climates, and higher teacher turnover (Branch et al., 2012; Grissom et al., 2021; Sebastian et al., 2016). In sum, investigating violence against administrators is critical to understanding and improving school functioning more broadly.

### 1.1. Theoretical Framework

The social ecological model offers a useful framework for examining violence against administrators. This framework takes into account the contexts in which individuals are nested, each with varying levels of interaction and mechanisms of reciprocal influence (Bronfenbrenner, 1976). Astor and Benbenishty (2019) have adapted the social ecological framework to understand school violence, conceptualizing a model where the school is the central figure, nested within multiple contexts. In addition to internal influences from students and teachers, external influences such as family, neighborhood, and societal characteristics impact school violence. School climate and cultural pluralism are important contextual dimensions to examine in relation to violence, as they reflect school norms and values, as well as interpersonal relationships within the school setting (Thapa et al., 2013). Further, administrators have different experiences of school climate than students, teachers, and staff (Capp et al., 2020), highlighting the necessity of assessing their perspectives. Thus, there is a need to examine influences at the individual, school, and community levels that affect violence and aggression against administrators.

#### 1.1.1. Student Strengths

Given that the central focus of schools is on the academic success of students, and as students contribute to a large portion of violence against school personnel (McMahon et al., 2024), it is helpful to consider student-level factors that contribute to violence against administrators. These may include student attendance, engagement in learning, student behaviors, and student–staff relationships. Student behaviors, such as aggressive and violent behaviors, may be influenced by students’ relationships with teachers and their sense of engagement and connection to the school (Johnson, 2009). Positive student–teacher relationships correlate negatively with school violence in the US and internationally (Y. Yang et al., 2021), and teacher support of students is associated with less student violence (Lesneskie & Block, 2017). Implementing small group activities and offering emotional and instructional support to students can facilitate behavioral engagement (Downer et al., 2007). Classroom engagement is also related to feelings of school safety (Côté-Lussier & Fitzpatrick, 2016). Further, feelings of connectedness lead to perceptions of more effective learning environments, which in turn protect against student aggressive and disruptive behaviors (Brookmeyer et al., 2006). Research is needed to explore student-level factors in relation to administrator-directed violence.

#### 1.1.2. School Strengths

School-level factors that ameliorate student violence in schools include consistent and fair school disciplinary practices, a positive school climate, and the inclusion of staff and student voices in determining school policies. Schools with consistent and fair disciplinary policies reported less student victimization and delinquent behavior (Gottfredson et al., 2005), lower rates of student classroom misbehavior (Welsh, 2003), lower levels of violent behavior and victimization (Johnson, 2009), and less teacher-directed violence (F. L. Huang et al., 2017). Additionally school staff perceptions of positive school climate are associated with lower levels of student violence and risk behaviors (Capp et al., 2020). An authoritative school climate, characterized by fair and strict disciplinary policies and support and respect for students, predicts less student aggression and greater school safety (Berg & Cornell, 2016). Little is known about how school-level factors influence administrator-directed violence.

#### 1.1.3. Community Strengths

Community-level factors, including parent–teacher relationships, district support and resources, and broader neighborhood and societal factors, may influence violence and aggression against administrators. Although research connecting community factors to administrator victimization is limited, these variables are associated with administrator well-being and retention (Perry et al., 2024). A national survey of school leaders conducted by the NASSP found that for high school administrators, active involvement in district-level decision-making and access to employer-provided mental health support are associated with better mental health and well-being (NASSP, 2023). Additionally, results from the survey indicated that greater societal respect, school funding, and professional development opportunities are reported by high school administrators as important factors that would enhance their likelihood to remain in their profession (NASSP, 2023). In addition, parent involvement in schools is associated with stronger relationships with teachers, greater trust in collaboratively addressing issues, and less overall school violence (Brookmeyer et al., 2006; Lesneskie & Block, 2017). Less overall school violence is likely related to lower rates of administrator-directed violence.

### 1.2. School Cultural Pluralism

Students often experience verbal and physical violence as a result of cultural tensions at their schools (Mansouri & Jenkins, 2010). Cultural pluralism refers to the coexistence and support of multiple distinct cultures within a society, emphasizing respect for cultural differences and fostering intergroup interactions (Bernstein, 2015). Some schools have incorporated cultural pluralism into their curricula and have adopted policies and practices to better serve diverse student populations and address intercultural conflicts when they occur. Within the school context, cultural pluralism incorporates acceptance and the celebration of cultural diversity by promoting culturally responsive teaching, multilingualism, and positive views of school diversity (Schachner et al., 2016). Studies show that school cultural pluralism is associated with better student outcomes (e.g., academic efficacy, achievement, quality of school life for minoritized students), and these findings are consistent across student-, teacher-, classroom-, and school-level reports (Brand et al., 2008). Cultural pluralism can facilitate students’ psychological school adjustment (Schachner et al., 2016), improve student–teacher relationships, and enhance student perceptions of fairness regarding school rules and policies (Smith et al., 2020). Additionally, students have favorable perceptions of the school climate when they view the school as supportive of cultural pluralism (Smith et al., 2020). Thus, schools’ cultural pluralism practices can have myriad benefits related to academics and the school climate.

There is also some evidence to suggest that cultural pluralism is associated with less school violence. For example, schools that implement cultural pluralism practices, such as cultural diversity acceptance groups, report less school violence regardless of the racial and ethnic composition of the school (Crawford & Burns, 2022). Cultural pluralism may shape school safety by creating environments where student, school, and community strengths can thrive. Indeed, multiculturalism in schools was shown to moderate the effectiveness of violence prevention programs, with outcomes suggesting that a culturally inclusive environment enhances the impact of these programs (Bardach et al., 2022). However, to date, studies have not explored the specific role of cultural pluralism in mitigating violence against school administrators. This study seeks to fill that gap by examining the potential protective influence of cultural pluralism, given its central role in shaping the broader school climate (Schachner et al., 2016).

### 1.3. Current Study

Although research on teacher-directed violence has increased significantly in the past 15 years (McMahon et al., 2024), research on administrator-directed violence remains scant. Given the high rates of violence (e.g., 70% reporting at least one incident; NASSP, 2023) and the critical role administrative leaders play in schools, research is needed to examine their experiences and the ecological context that contributes to these experiences. School administrators are uniquely positioned to report on ecological strengths and cultural pluralism practices, as well as facilitate improvements in school functioning and safety. This study addresses several gaps in the literature, including the limited research on school administrators’ perspectives, the predominant focus on risk factors over strength-based frameworks, and the lack of attention paid to cultural context. Accordingly, this study aims to examine the role of ecological strengths and cultural pluralism in predicting different types of administrator-directed violence from student, parent, and colleague offenders. Specifically, we have three research questions: (1) To what extent do administrator experiences of verbal/threatening and physical violence across offenders vary by demographics at the individual (e.g., race/ethnicity), school (e.g., school level), and community (e.g., urbanicity) levels? (2) Are greater strengths at the student, school, and community level associated with lower rates of verbal/threatening and physical violence against administrators? (3) Do school cultural pluralism practices moderate the association between ecological strengths at the student, school, and community level and administrator victimization (i.e., verbal/threatening and physical violence)?

## 2. Method

### 2.1. Participants

Respondents were 497 pre-K–12th grade school administrators from 42 states who participated in the spring of 2022 as part of a larger study directed by the APA Task Force on Violence Against Educators and School Personnel (McMahon et al., 2024). More than half of the sample self-identified as female (*n* = 293; 59%), followed by male (*n* = 199; 40%), with five participants (1%) not responding. The majority of the sample self-identified as White/Caucasian (*n* = 367; 73.8%), followed by Black/African American (*n* = 62; 12.5%), Hispanic/Latinx (*n* = 39; 7.8%), multiracial (*n* = 17; 3.4%), Asian (*n* = 5; 1%), and other race (*n* = 3; 0.6%), with 4 participants (0.8%) not providing information on race/ethnicity. Participants served in a variety of school administrative roles including principal or head of school (*n* = 182; 36.6%), assistant or vice principal (*n* = 171; 34.4%), district-level administrator (*n* = 68; 13.7%), other administrative role (*n* = 39; 7.8%), dean of students (*n* = 24; 4.8%), building administrator (*n* = 12; 2.4%), and dean of faculty (*n* = 1; 0.2%). Participants worked in elementary schools (*n* = 185; 37.2%), high schools (*n* = 114; 22.9%), pre-K–12th grade schools (*n* = 92; 18.5%), and middle schools (*n* = 77; 15.5%) with 29 participants (5.8%) not reporting their school level. Respondents worked in various settings, including suburban (*n* = 195; 39%), urban (*n* = 169; 34%), and rural (*n* = 133; 27%) schools. On average, respondents had nine years of experience in their field (*SD* = 7.33). The gender, race/ethnicity, and years of experience of administrators in our sample are comparable with national demographics (NCES, 2023a).

### 2.2. Measures

#### 2.2.1. Ecological Strengths and Problems Scale

The Ecological Strengths and Problems Scale (McMahon et al., 2025) asked participants, on a scale of 1 to 5, how much a series of factors were a problem versus a strength at their school during this school year (1 = *significant problem*; 2 = *slight problem*; 3 = *neither a problem nor a strength*; 4 = *slight strength*; 5 = *significant strength*). A total of 16 items made up three unique factors—student (5 items: student attendance, students engaged in learning, students followed rules, student use of internet/social media, student–staff relationships; α = 0.79, ω = 0.81), school (4 items: school-level administrative support, school climate, staff voice in school policies and practices, school disciplinary practices; α = 0.82, ω = 0.82), and community (7 items: parent/guardian engagement, attention to diversity and inclusion, special education services, school district supports, parent–teacher relationships, school neighborhood characteristics, societal factors; α = 0.80, ω = 0.80)—with each subscale yielding a mean score. Model fit was affirmed for the scale via confirmatory factor analysis (CFA) with weighted least square mean and variance-adjusted estimation (WLSMV; root mean square of approximation [RMSEA] = 0.09 [90% CI: 0.08–0.10]; comparative fit index [CFI] = 0.95; Tucker–Lewis index [TLI] = 0.93; chi-square [χ^2^] = 5182.47 (*df* = 120, *p* < 0.001); standardized root mean square residual [SRMR] = 0.05). In performing the CFAs, correlated errors were allowed for a single pair of items on each of the three factors for acceptable fit due to cross-loading. We opted to allow these correlated errors to preserve the original set of items.

#### 2.2.2. Administrator Victimization

The Educator Victimization Scale (McMahon et al., 2024) assessed administrator experiences with violence and included ten items with two subscales: *verbal/threatening* (7 items: obscene remarks or gestures, slurs or verbal attacks based on demographic characteristics [e.g., ethnicity], verbal threats, intimidation, public humiliation, cyber or internet bullying, bullying) and *physical violence* (3 items: objects thrown, an ordinary object used as a weapon, physically attacked). Administrators were asked to rate how often they experienced each type of violent behavior from student, parent, and colleague aggressors during the school year. Response options were on a 6-point Likert-type scale (0 = *never*, 1 = *once*, 2 = *a few times*, 3 = *monthly*, 4 = *weekly*, 5 = *daily*). Mean subscale scores were created. Reliability estimates were calculated for each factor, including verbal/threatening violence from students (α = 0.82, ω = 0.84), parents (α = 0.82, ω = 0.82), and colleagues (α = 0.78, ω = 0.78), and physical violence from students (α = 0.84, ω = 0.88), parents (α = 0.89, ω = 0.90), and colleagues (α = 0.82, ω = 0.85).

CFAs with WLSMV estimation were conducted on the item sets as three separate two-factor models (verbal/threatening and physical) of administrator-directed violence, with violence by student (RMSEA = 0.05 [90% CI: 0.03–0.07], CFI = 0.99, TLI = 0.99, χ^2^ = 2783.39 (*df* = 45, *p* < 0.001), SRMR = 0.04), parent (RMSEA = 0.07 [90% CI: 0.05–0.09], CFI = 0.98, TLI = 0.97, χ^2^ = 2434.82 (*df* = 45, *p* < 0.001), SRMR = 0.08), and colleague (RMSEA = 0.05 [90% CI: 0.03–0.07], CFI = 0.98, TLI = 0.97, χ^2^ = 1471.63 (*df* = 45, *p* < 0.001), SRMR = 0.08) aggressors examined separately, and all items exhibiting good model fit.

#### 2.2.3. School Cultural Pluralism

Perceptions of respect and support for cultural pluralism during the school year were measured via a four-item scale, with one item drawn from a subscale of the Inventory for School Climate (Brand et al., 2008) and three items developed by the APA Task Force on Violence Against Educators and School Personnel for this study. The measure asks participants to rate their level of agreement with four items on a Likert-type scale (1 = *Strongly disagree*, 2 = *Disagree*, 3 = *Neither agree nor disagree*, 4 = *Agree*, 5 = *Strongly agree*); these items include “students in this school respect each other’s cultural differences”, “school staff show that they think it is important for students of different cultures, identities, and demographic groups at this school to get along with each other”, “school staff honor student voice and culture in the curriculum and discussion”, and “there is a lot of tension in this school between people of different cultures, identities, and demographic groups” (reverse-scored) (α = 0.75, ω = 0.74). A mean scale score was computed. CFA with maximum likelihood estimation confirmed that model fit for this scale was acceptable, except for an inflated RMSEA value (RMSEA = 0.15 [90% CI: 0.09–0.21], CFI = 0.99, TLI = 0.99, χ^2^ = 3421.36 (*df* = 6, *p* < 0.001), SRMR < 0.01). We chose to retain this model despite the elevated RMSEA value as RSMEA estimates fluctuate as a function of model complexity and degrees of freedom, and modifications to models with high RMSEA values in conjunction with excellent values for other model fit indices are not recommended (McNeish, 2018).

### 2.3. Procedure

Following Institutional Review Board (IRB) procedures approved at University of North Carolina at Chapel Hill, data were collected via an online survey that was administered by the APA Task Force on Violence Against Educators and School Personnel from March to June 2022. School participants were contacted via school emails provided by a national marketing firm (MCH Strategic Data) and through national partners. MCH Strategic Data gathers and updates teacher contact information by conducting website scans of public education data sources and importing this information into a comprehensive national database of school staff. Participants were provided with a link to the online survey describing the study’s purpose and IRB-approved informed consent. Participant data used in this study were de-identified.

### 2.4. Data Analytic Plan

First, descriptive statistics were examined, and rates of verbal/threatening and physical violence were calculated by offender (i.e., student, parent, colleague). Second, a multivariate analysis of variance (MANOVA) was run using SPSS version 29 to assess mean differences in verbal/threatening and physical violence by offender (i.e., student, parent, colleague) across demographic characteristics (i.e., gender, race/ethnicity, years of experience, school level, and urbanicity). Third, using Mplus Version 8, a path analysis with composite scores based on means was conducted with ecological strengths (student, school, and community), predicting verbal/threatening and physical violence by student, parent, and colleague aggressors. The main effect of cultural pluralism on administrator victimization was also included. This model was then re-run with three additional terms, which represented the centered interactions between cultural pluralism and student, school, and community strengths, to predict administrator victimization. A Johnson–Neyman analysis and follow-up simple slopes analysis were conducted to probe the significant interaction. For both analyses, covariates were respondents’ years of experience, gender, race/ethnicity (i.e., White/Caucasian, Black/African American, Hispanic/Latinx, Multiracial/Other), school level (e.g., middle school, high school, pre-K–12th grade schools), and school urbanicity (i.e., rural, suburban, and urban). Categorical covariates were dummy-coded such that female (for gender), White/Caucasian (for race/ethnicity), elementary schools (for school level), and urban (for urbanicity) were all selected as reference categories. For both the main effects model and the moderation model, all demographic covariates were not significantly associated with any outcome variables, so they were trimmed, and the model was re-run without them. This model trimming exercise was performed to ensure model fit was generated for the two primary analytic models.

## 3. Results

### 3.1. Descriptive Statistics and Rates of Violence

**Figure 1:**
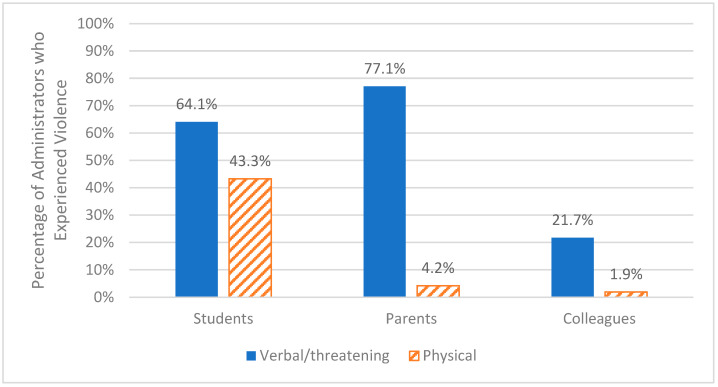
Violence against administrators by aggressor. *Note:* responses were dichotomized based on participants indicating they experienced no violence versus at least one incident of violence during the school year.

Descriptive statistics, including correlations, are presented in Table 1. Overall, 87.4% of administrators reported at least one incident of verbal/threatening violence, and 44.8% reported at least one incident of physical violence. Rates of violence varied depending on the aggressor and type of violence (see Figure 1). The most commonly experienced type of violence was verbal/threatening violence from parents, with more than three-quarters of administrators reporting at least one incident during the school year. This was followed by verbal/threatening (64%) and physical violence (43%) from students, followed by over one-fifth of the sample reporting verbal/threatening violence from colleagues. Fewer than 5% of administrators reported physical violence from parents or colleagues. In general, student, school, and community strengths were associated with less verbal/threatening violence from students and parents, less physical violence from students, and greater school cultural pluralism. Ecological strengths were not associated with parent or colleague physical violence. Cultural pluralism was significantly correlated with less verbal/threatening violence from students, parents, and colleagues, and less physical violence from students.

### 3.2. Demographic Differences in Administrator Victimization

Demographic covariates (i.e., gender, race/ethnicity, years of experience, school level, and urbanicity) were entered into a MANOVA to assess mean differences in self-reported violence across demographic characteristics. According to the multivariate omnibus tests, race/ethnicity (Pillai’s Trace = 0.17, *F*(30, 1290) = 1.50, *p =* 0.04) and school level (Pillai’s Trace = 0.45, *F*(18, 768) = 7.43, *p <* 0.01) were both significantly related to the outcome variables. An examination of the between-group effects showed that the significant effect of race/ethnicity was seen for physical violence from parents (*F*(5, 259) = 2.62. *p* = 0.03), while the significant effect of school level was seen for verbal/threatening violence from students (*F*(3, 259) = 4.55. *p* < 0.01) and physical violence from students (*F*(3, 259) = 19.52. *p* < 0.01). Pairwise comparisons indicated that Asian administrators experienced more physical violence from parents compared to White (*β* = −0.62, *p* < 0.01) and Latinx (*β* = −0.64, *p* < 0.01) participants. Further, participants from pre-K–12th grade schools experienced significantly less verbal violence from students compared to participants in middle (*β* = 0.40, *p* < 0.01) and high schools (*β* = 0.42, *p* < 0.01). Finally, elementary school administrators experienced significantly more physical violence from students compared to participants from any other school level (middle schools: *β* = −0.63, *p* < 0.01; high schools: *β* = −0.87, *p* < 0.01; preK-12th grade schools: *β* = −0.74, *p* < 0.01).

### 3.3. Initial Model Results with Main Effects Only (No Moderation)

**Figure 2:**
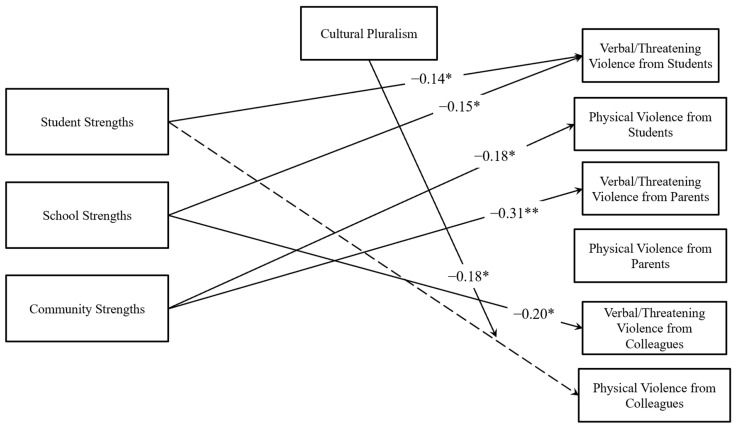
Path analysis results. *Notes: N* = 497. * *p* < 0.05. ** *p* < 0.01. Solid lines represent statistically significant main effects. Dotted lines represent non-statistically significant main effects that were significantly moderated by cultural pluralism. Non-statistically significant and unmoderated paths, along with relations for demographic variables, are not presented for figure clarity. All reported coefficients are standardized estimates. The main effect statistics reported here are from the main effects path analysis, while the interaction terms are from the moderation path analysis.

Student, school, and community strengths were entered as predictors of verbal/threatening and physical violence against administrators in a path analysis using Mplus Version 8 (see Table 2 and Figure 2). The main effect of cultural pluralism was also included prior to moderation analyses. The model showed excellent model fit (RMSEA = 0.04 [90% CI: 0.01–0.05], CFI = 0.98, TLI = 0.93, χ^2^ = 38.34 (*df* = 24, *p* = 0.03), SRMR = 0.03). The percentages of variance explained in the outcome variables were as follows: verbal violence from students—13.1%; physical violence from students—6.3%; verbal violence from parents—10.9%; physical violence from parents—1.5%; verbal violence from colleagues—3.6%; and physical violence from colleagues—0.8%.

Results indicated that student strengths were negatively related to verbal/threatening violence from students, meaning that the more that administrators endorsed student strengths, the less verbal/threatening violence they experienced from students. School-level strengths were negatively related to verbal/threatening violence from students and colleagues; when participants endorsed greater school strengths, they experienced less violence. Community strengths were also negatively related to physical violence from students and verbal/threatening violence from parents, indicating greater community strengths were associated with less violence. Although cultural pluralism was significantly correlated with verbal violence from students, parents, and colleagues, as well as physical violence from students, cultural pluralism did not significantly predict any type of violence from any offender as a main effect in the model. Similarly, while race/ethnicity and school level were significant predictors of certain types of violence in the MANOVA, demographic variables were not significant predictors of violence in the path analysis when student, school, and community effects were taken into account.

### 3.4. Model Results with Cultural Pluralism Practices Moderator

We created the same model in SPSS version 29 and it was re-run, employing the three centered interaction terms between cultural pluralism and student, school, and community factors to predict administrator violence. Only the interaction terms are reported (see Table 2 and Figure 2). This model also showed excellent fit with the data (RMSEA = 0.03 [90% CI: 0.00–0.04], CFI = 0.99, TLI = 0.96, χ^2^ = 46.15 (*df* = 35, *p* = 0.10), SRMR = 0.02). The percentages of variance explained in the outcome variables were as follows: verbal violence from students—17.4%; physical violence from students—25.0%; verbal/threatening violence from parents—14%; physical violence from parents—2.9%; verbal/threatening violence from colleagues—4.8%; physical violence from colleagues—3.9%.

**Figure 3:**
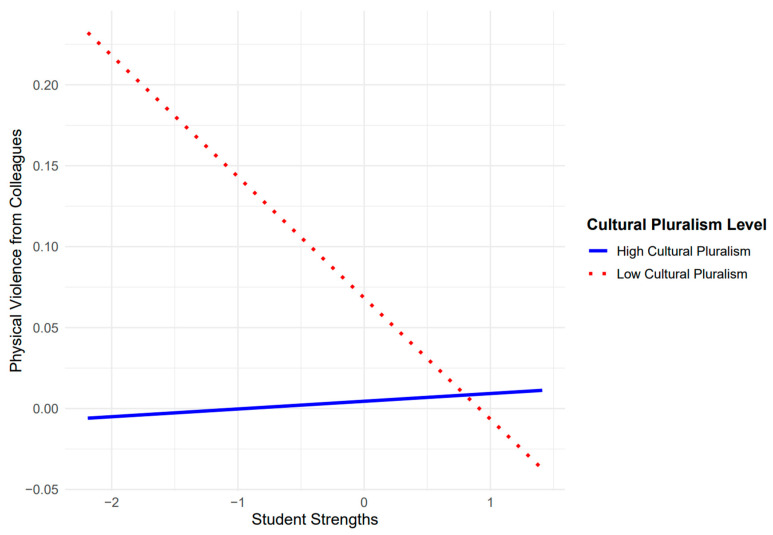
Effect of student strengths on physical violence from colleagues at different levels of cultural pluralism. Note: Cultural pluralism was categorized into low (one standard deviation below the mean) and high (one standard deviation above the mean) groups. Both the student strengths and cultural pluralism variables were mean-centered to facilitate the interpretation of the interaction.

Cultural pluralism was a significant moderator of the association between student strengths and physical violence from colleagues (see Figure 3). The Johnson–Neyman analysis revealed that the relationship between student strengths and physical violence from colleagues was significant at low levels of cultural pluralism (cultural pluralism < −0.12, *p* < 0.05), but not significant at average or higher levels of cultural pluralism (cultural pluralism ≥ −0.12) within the observed range of data (−2.71 to 1.29). To further examine the interaction, simple slopes analyses were conducted at three levels of cultural pluralism (−1 SD [low], the mean, and +1 SD [high]). At low cultural pluralism (*M* = −0.77), student strengths were negatively related to physical violence from colleagues (*B* = −0.05, *p* < 0.001). At average values of cultural pluralism (*M* = −0.03), the slope of student strengths on physical colleague violence was marginally negative (*B* = −0.02, *p* = 0.08), suggesting a weaker, but still negative relationship that was not statistically significant at the conventional threshold. At high values of cultural pluralism (*M* = 0.72), there was no significant relationship between student strengths and physical violence from colleagues (*B* = 0.00, *p* = 0.82).

It is worth pointing out that several of the interaction effects approached statistical significance (e.g., *p* = 0.07–0.09) in relation to administrator victimization and contributed to small increases in the explained variance in the outcome variables (β = 0.14–0.19). These interactions and the role of cultural pluralism are worthy of investigation in future studies.

## 4. Discussion

This investigation is one of the few studies examining violence against school administrators. Using a social ecological framework emphasizing strengths, this study uniquely examined the direct and contextual factors that predict administrator-directed violence in the US. Violence against administrators is common, and in the current study, we found that 87.4% of administrators reported experiencing verbal/threatening violence, and nearly 45% experienced physical violence during the school year. These findings suggest the rates are similar or higher compared to previous US and international studies (Arnold et al., 2021; McMahon et al., 2024). It is notable that the highest rates of verbal/threatening violence against administrators were from parents. This differs from teacher-directed violence studies, which show teachers experience the most verbal/threatening violence from students (e.g., McMahon et al., 2024). Findings from this study demonstrate that strengths at the student, school, and community levels are associated with fewer experiences of violence among administrators; however, these associations vary by type of violence and aggressor. Cultural pluralism moderated the relation between student strengths and physical violence from colleagues, highlighting the complex role of cultural pluralism in schools.

### 4.1. To What Extent Does Administrator Victimization Vary by Individual, School, and Community Characteristics?

Our study identified significant differences in the experiences of violence based on administrator race/ethnicity and school level. Although there were few Asian administrators in this sample, those who identified as Asian reported experiencing more physical violence from parents compared to those who identified as White or Latinx. These findings contribute to a complex and somewhat mixed body of literature on race/ethnicity-based differences in violence against educators. For example, prior research has found that teachers from ethnically (Wei et al., 2013) and racially (NCES, 2023b) underrepresented backgrounds report higher rates of verbal and physical violence than their White counterparts. However, other studies have found that teachers who identify as racial or ethnic minorities are less likely to report certain types of violence; these differences may be due to factors such as different cultural understandings of and adaptations to violence (McMahon et al., 2014). Overall, more research is needed in this area, as school administrators’ workplace safety concerns may differ from those of teachers.

We found that elementary school administrators reported more physical violence from students than those at any other level. This aligns with previous research on teachers, which has shown that verbal aggression peaks in middle and high school, whereas physical aggression is most common in elementary school (Bounds & Jenkins, 2018; McMahon et al., 2022b). These patterns are often attributed to developmental changes, including increased relational aggression in older students due to evolving social dynamics and language skills, as well as younger children’s lower capacity to regulate emotions and behaviors. Given that administrators in pre-K–12th grade schools reported lower levels of verbal/threatening violence from students compared to those in middle and high schools, closer examination of what other characteristics might be playing a role (e.g., pre-K–12 schools possibly more likely to be smaller, private, religious, or in rural settings) is necessary. Overall, our findings contribute to the limited body of literature on demographic differences across individual, school, and community levels in administrator-directed violence.

### 4.2. Are Student, School, and Community Strengths Associated with Administrator Victimization?

Results demonstrated that student strengths (e.g., positive student behavior, high levels of engagement, attendance, and rule-following, positive relations with staff) were associated with less verbal and threatening violence from students against administrators. This finding is consistent with previous research indicating that greater student and classroom engagement (Bradshaw et al., 2021; C. Yang et al., 2018), student rule adherence (Fisher et al., 2018), positive student–staff relationships (Krause & Smith, 2022; Wang et al., 2015), and fewer student behavioral issues (Lindstrom Johnson et al., 2017) are associated with greater school safety or less school violence. Our research highlights how student strengths like engagement and rule-following can be impactful, extending previous findings on school violence and school safety to include lower rates of violence against administrators.

Similarly, greater school-level strengths (e.g., fair disciplinary practices, positive school climate, administrative support, staff voice) were associated with less verbal/threatening violence from students and colleagues. Previous research has demonstrated that a negative school climate is associated with student violent or aggressive behavior against teachers (F. Huang & Anyon, 2020). Similarly, an authoritative school climate characterized by structure and support, including consistent enforcement of school rules, contributes to less violence against educators (Kapa et al., 2018). The current study extends these findings to administrators as targets of violence and broadens the focus from student aggressors to also include colleague aggressors. These findings may also suggest that school-level forms of informal social control, such as administrative support and promoting staff voices, may lead to fewer issues among students and colleagues, thus leading to less violence.

Community-level strengths were related to less verbal and threatening violence from parents, indicating that administrators experienced less violence from parents when they perceived greater strength in areas such as parent–teacher relationships, parent engagement, and community safety. Parent involvement in school events, volunteering, and academics is protective against general school violence and in the relationship between neighborhood crime and school violence (Song et al., 2019). Improved parent–teacher relationships are also related to less teacher-directed violence from parents in Italy (De Cordova et al., 2019). Community-level strengths were also associated with less physical violence from students. It is possible that factors such as positive relationships with parents and school district support allow for better communication, interventions, and resources to prevent the escalation of student violence (Zulauf-McCurdy & Zinsser, 2022). More research is needed on strengths across ecological levels of administrator-directed violence.

### 4.3. Does Cultural Pluralism Moderate the Relation Between Ecological Strengths and Administrator Victimization?

The relationship between student strengths and physical violence from colleagues varied as a function of cultural pluralism. Specifically, student strengths were negatively associated with colleague physical violence at low levels of cultural pluralism, and this relationship became marginally significant and nonsignificant at average and high levels of cultural pluralism, respectively. This suggests that, in environments with lower cultural pluralism, student strengths (e.g., attendance, engagement in learning, and positive relationships with staff) play a more critical role in influencing physical violence from colleagues than when cultural pluralism is moderate or high. Perhaps in environments with weaker norms of inclusivity and harmony across cultural groups, student strengths may help foster a more positive school climate, thereby reducing workplace violence. However, in schools with moderate to high levels of cultural pluralism, student strengths do not significantly predict physical violence from colleagues, indicating that other contextual factors may be more influential in these settings. That is, cultural pluralism is especially beneficial in reducing colleague violence against administrators when student strengths are low. It is possible that in schools with greater cultural pluralism, broader institutional or cultural factors contribute to a more cohesive environment, diminishing the role of student-level characteristics in shaping workplace dynamics.

The literature on violence against school administrators is largely limited to prevalence rates; however, research on school violence more broadly aligns with our findings. Specifically, positive interpersonal relationships among school staff and faculty have been linked to lower levels of workplace violence (e.g., excessive workload, ridicule, physical abuse) and reduced experiences of discrimination (e.g., wages, benefits, and performance evaluations; Kim & Ko, 2022). Further, prior research suggests that cultural pluralism is associated with lower levels of interpersonal violence among students (Le & Johansen, 2011). Although we did not find a direct effect of cultural pluralism on violence in our model examining ecological strengths, our findings indicate cultural pluralism is associated with less administrator violence and our moderator findings indicate that cultural pluralism changes the relationship between student strengths and colleague violence. Further research is needed to clarify the mechanisms through which cultural pluralism interacts with student behaviors and staff dynamics to promote safer workplaces.

Cultural pluralism was not a significant moderator in the relationship between student strengths and violence from students or parents, or in the relationship between school or community strengths and any forms of violence. It is possible that student, school, and community strengths may play a more central and consistent role in predicting violence against administrators, regardless of cultural pluralism, but it is also possible that the role of cultural pluralism varies based upon the cultural differences or homogeneity of the school. Principles that embrace respect for and celebration of cultural differences may foster a positive school climate with the potential to reduce violence. Future research should explore cultural differences, cultural dynamics, and the role of cultural pluralism in school violence and its influence on violence against students, teachers, staff, and administrators.

### 4.4. Limitations

This study has several limitations. First, the design is cross-sectional, so neither causal effects nor trends over time can be determined. Second, violence from colleague aggressors, especially physical violence, was low and skewed, and the skewness may have affected the reliability of the correlation coefficients. Third, it is possible that administrators who responded to this survey were more likely to have experienced violence in their schools. Despite these limitations, the path analyses employed full information maximum likelihood to estimate missing data, eliminating the need for listwise deletion and thus optimizing all available data. This study’s strength-based approach with an understudied population yields implications for schools to build upon strengths and reduce violence across the school ecology.

### 4.5. Implications for Research, Practice, and Policy

There is a need for improved methodological design in educator- and school personnel-directed violence studies, particularly in the form of longitudinal and multi-informant research. Research is also needed to investigate the impacts of violence on administrators, including how victimization may affect stress, mental health, job dissatisfaction, and attrition (Perry et al., 2024). Given the few studies on administrator-directed violence, qualitative and mixed-method research may facilitate an understanding of these complex relationships. The current findings indicate that ecological strengths are associated with less violence, but further investigation of classroom and school practices, policies, and training, as well as the mechanisms driving the moderating effect of cultural pluralism, would facilitate intervention development.

In terms of school practice and policy, support and respect for cultural pluralism may be instrumental in strengthening the overall school ecology, which may contribute to violence reduction in schools. Further, creatively engaging students in learning, developing student–staff relationships, enhancing administrative support, improving school climate, and incorporating staff voice in school policies and practices may be helpful practices in reducing violence. In addition, an annual assessment of violence experienced across school stakeholders, including administrators, as well as their training needs and recommendations will facilitate assessment and intervention.

## 5. Conclusions

Research on violence against school administrators is limited, and this study bridges that gap by assessing the prevalence and correlates of administrator-directed violence from students, parents, and colleagues. Demographic findings reveal differences by race/ethnicity and school level. Greater ecological strengths at the student, school, and community levels were associated with less verbal/threatening and physical violence against administrators by multiple offenders. Cultural pluralism also played an important role, moderating the relation between student strengths and colleague violence. Thus, it may be especially important to highlight the need to uplift and honor diverse voices and cultures, reduce cultural tensions, and incorporate student voice and cultures into the curriculum when student strengths are low. This study suggests multiple strategies schools can implement to support the safety and well-being of administrators.

## Figures and Tables

**Table 1:** Descriptive statistics and correlations of school administrator-reported scales.

	*M*	*SD*	1	2	3	4	5	6	7	8	9	10
1. Student strengths	2.39	0.89	-									
2. School strengths	3.18	1.00	0.56 **	-								
3. Community strengths	2.86	0.79	0.60 **	0.69 **	-							
4. Cultural pluralism	3.70	0.74	0.35 **	0.48 **	0.44 **	-						
5. Verbal/threatening violence from students	0.55	0.71	−0.32 **	−0.33 **	−0.31 **	−0.23 **	-					
6. Physical violence from students	0.61	0.93	−0.08	−0.16 **	−0.19 **	−0.11 *	0.54 **	-				
7. Verbal/threatening violence from parents	0.69	0.68	−0.16 **	−0.21 **	−0.31 **	−0.16 **	0.52 **	0.43 **	-			
8. Physical violence from parents	0.06	0.34	0.02	−0.04	−0.01	−0.03	0.21 **	0.24 **	0.38 **	-		
9. Verbal/threatening violence from colleagues	0.16	0.39	−0.06	−0.16 **	−0.10	−0.11 *	0.30 **	0.20 **	0.38 **	0.41 **	-	
10. Physical violence from colleagues	0.03	0.19	0.05	0.003	0.04	−0.06	0.15 **	0.10	0.19 **	0.60 **	0.50 **	-

*Note. N* = 497. * *p* < 0.05. ** *p* < 0.01. Covariates were not included for table simplicity.

**Table 2:** Path Analysis Results.

**DV: Verbal/threatening violence from students**	**β**	***SE***	***p***
Student strengths	−0.14	0.07	0.05
School strengths	−0.15	0.08	0.05
Community strengths	−0.07	0.08	0.37
Cultural pluralism	−0.04	0.06	0.55
Student strengths × cultural pluralism	0.10	0.08	0.21
School strengths × cultural pluralism	0.09	0.09	0.32
Community strengths × cultural pluralism	−0.14	0.08	0.08
**DV: Physical violence from students**			
Student strengths	0.08	0.07	0.25
School strengths	−0.07	0.08	0.37
Community strengths	−0.18	0.08	0.03
Cultural pluralism	−0.002	0.07	0.97
Student strengths × cultural pluralism	0.05	0.08	0.56
School strengths × cultural pluralism	0.09	0.09	0.30
Community strengths × cultural pluralism	−0.14	0.08	0.09
**DV: Verbal/threatening violence from parents**			
Student strengths	0.07	0.07	0.28
School strengths	−0.02	0.08	0.83
Community strengths	−0.31	0.08	<0.01
Cultural pluralism	−0.06	0.06	0.31
Student strengths × cultural pluralism	−0.11	0.08	0.17
School strengths × cultural pluralism	0.17	0.09	0.07
Community strengths × cultural pluralism	−0.01	0.08	0.89
**DV: Physical violence from parents**			
Student strengths	0.09	0.08	0.22
School strengths	−0.14	0.09	0.10
Community strengths	0.002	0.09	0.98
Cultural pluralism	0.03	0.07	0.65
Student strengths × cultural pluralism	−0.11	0.09	0.22
School strengths × cultural pluralism	−0.02	0.11	0.88
Community strengths × cultural pluralism	−0.002	0.09	0.99
**DV: Verbal/threatening violence from colleagues**			
Student strengths	0.06	0.07	0.43
School strengths	−0.20	0.08	0.02
Community strengths	0.01	0.08	0.88
Cultural pluralism	−0.03	0.07	0.69
Student strengths × cultural pluralism	−0.10	0.09	0.25
School strengths × cultural pluralism	0.19	0.10	0.07
Community strengths × cultural pluralism	−0.07	0.09	0.95
**DV: Physical violence from colleagues**			
Student strengths	0.08	0.07	0.26
School strengths	−0.08	0.09	0.33
Community strengths	0.05	0.09	0.59
Cultural pluralism	−0.04	0.07	0.58
Student strengths × cultural pluralism	−0.18	0.09	0.04
School strengths × cultural pluralism	0.05	0.10	0.63
Community strengths × cultural pluralism	−0.05	0.09	0.59

*Note. N* = 497. DV = dependent variable. Coefficients for covariates are not presented for table clarity. The main effect statistics reported here are from the main effects path analysis, while the interaction terms are from the moderation path analysis.

## Data Availability

The data are not currently available for public access due to the size of the data sets, which are currently undergoing ongoing data cleaning, organizing, scale refinement, and scale validation. The study materials are available upon request.
